# Diaqua­bis(1,3-propane­diamine)nickel(II) squarate tetrahydrate

**DOI:** 10.1107/S160053680902087X

**Published:** 2009-06-06

**Authors:** Ersin Temel, Hakan Erer, Okan Zafer Yeşilel, Orhan Büyükgüngör

**Affiliations:** a Department of Physics, Faculty of Arts and Sciences, Ondokuz Mayıs University, Kurupelit, TR-55139, Samsun, Turkey; b Department of Chemistry, Faculty of Arts and Sciences, Eskişehir Osmangazi University, TR-26480, Eskişehir, Turkey

## Abstract

The asymmetric unit of the title compound, [Ni(C_3_H_10_N_2_)_2_(H_2_O)_2_](C_4_O_4_)·4H_2_O, contains one-half of the diaqua­bis(1,3-propane­diamine)nickel(II) cation, one-half of the centrosymmetric squarate anion and two uncoordinated water mol­ecules. In the cation, the Ni^II^ atom is located on a crystallographic inversion centre and has a slightly distorted octa­hedral coordination geometry. The six-membered chelate ring adopts a chair conformation. O—H⋯O hydrogen bonds link the cation and anion through the water mol­ecule, while N—H⋯O hydrogen bonds link the cation and anion and cation and water mol­ecules. In the crystal structure, inter­molecular O—H⋯O and N—H⋯O hydrogen bonds link the mol­ecules into a three-dimensional network structure.

## Related literature

For general background, see: Bertolasi *et al.* (2001 ▶); Gollogly & Hawkins (1972 ▶); Lam & Mak (2000 ▶); Liebeskind *et al.* (1993 ▶); Mathew *et al.* (2002 ▶); Reetz *et al.* (1994 ▶); Seitz & Imming (1992 ▶); Zaman *et al.* (2001 ▶). For related structures, see: Ghosh *et al.* (1997 ▶); Mukherjee *et al.* (1990 ▶); Pariya *et al.* (1995 ▶). For ring-puckering parameters, see: Cremer & Pople (1975 ▶). For bond-length data, see: Allen *et al.* (1987 ▶).
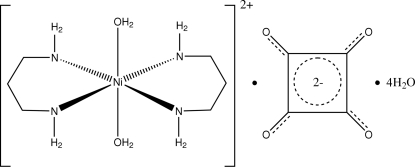


## Experimental

### 

#### Crystal data

[Ni(C_3_H_10_N_2_)_2_(H_2_O)_2_](C_4_O_4_)·4H_2_O
*M* *_r_* = 427.09
Monoclinic, 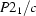

*a* = 8.0429 (4) Å
*b* = 9.1752 (5) Å
*c* = 14.6510 (8) Å
β = 117.570 (4)°
*V* = 958.40 (9) Å^3^
*Z* = 2
Mo *K*α radiation
μ = 1.06 mm^−1^
*T* = 296 K
0.75 × 0.45 × 0.05 mm

#### Data collection

Stoe IPDS II diffractometer
Absorption correction: integration (*X-RED32*; Stoe & Cie, 2002 ▶) *T*_min_ = 0.638, *T*_max_ = 0.949
7288 measured reflections
2204 independent reflections
2003 reflections with *I* > 2σ(*I*)
*R*_int_ = 0.020

#### Refinement

*R*[*F* ^2^ > 2σ(*F* ^2^)] = 0.021
*wR*(*F* ^2^) = 0.054
*S* = 1.06
2204 reflections
139 parameters
H atoms treated by a mixture of independent and constrained refinement
Δρ_max_ = 0.29 e Å^−3^
Δρ_min_ = −0.17 e Å^−3^

### 

Data collection: *X-AREA* (Stoe & Cie, 2002 ▶); cell refinement: *X-AREA*; data reduction: *X-RED32* (Stoe & Cie, 2002 ▶); program(s) used to solve structure: *SHELXS97* (Sheldrick, 2008 ▶); program(s) used to refine structure: *SHELXL97* (Sheldrick, 2008 ▶); molecular graphics: *ORTEP-3 for Windows* (Farrugia, 1997 ▶); software used to prepare material for publication: *WinGX* (Farrugia, 1999 ▶).

## Supplementary Material

Crystal structure: contains datablocks I. DOI: 10.1107/S160053680902087X/hk2703sup1.cif

Structure factors: contains datablocks I. DOI: 10.1107/S160053680902087X/hk2703Isup2.hkl

Additional supplementary materials:  crystallographic information; 3D view; checkCIF report

## Figures and Tables

**Table:** 

O1—Ni1	2.1429 (9)
N1—Ni1	2.1090 (10)
N2—Ni1	2.0997 (10)

**Table:** 

N1—Ni1—O1	88.86 (4)
N2—Ni1—O1	91.46 (4)
N2—Ni1—N1	91.94 (4)

**Table 2:** Hydrogen-bond geometry (Å, °)

*D*—H⋯*A*	*D*—H	H⋯*A*	*D*⋯*A*	*D*—H⋯*A*
N1—H1*B*⋯O5^i^	0.90	2.35	3.1715 (16)	152
N2—H2*A*⋯O2^ii^	0.90	2.33	3.2174 (14)	170
O1—H1*F*⋯O2^iii^	0.77 (2)	2.08 (2)	2.8345 (15)	165 (2)
O4—H4*A*⋯O2^iii^	0.80 (2)	2.10 (2)	2.8765 (16)	165 (2)
O4—H4*B*⋯O2^iv^	0.82 (3)	2.07 (3)	2.8965 (16)	178 (2)
O5—H5*B*⋯O4^v^	0.77 (2)	2.10 (3)	2.8730 (19)	177 (2)
N1—H1*A*⋯O4	0.90	2.38	3.2442 (17)	160
N2—H2*B*⋯O3	0.90	2.04	2.9333 (14)	174
O1—H1*E*⋯O5	0.80 (2)	1.93 (2)	2.7311 (16)	175.8 (19)
O5—H5*A*⋯O3	0.80 (2)	1.94 (2)	2.7296 (16)	166 (2)

Symmetry codes: (i) 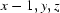; (ii) 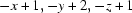; (iii) 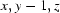; (iv) 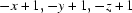; (v) 
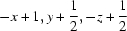
.

**Fig. 1.**
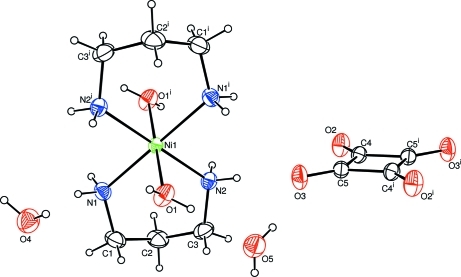
The molecular structure of the title molecule, with the atom-numbering scheme. Displacement ellipsoids are drawn at the 40% probability [symmetry code: (i) 1 - x, 1 - y, 1 - z.

**Fig. 2.**
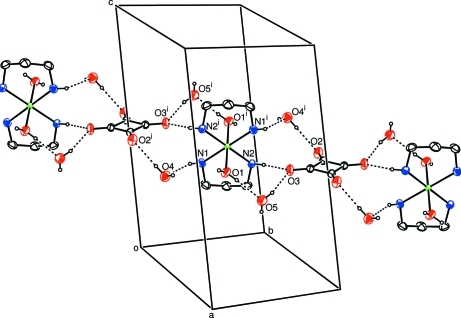
A partial packing diagram of the title compound. Hydrogen bonds are shown as dashed lines. H atoms not involved in hydrogen bonding have been omitted for clarity [symmetry code: (i) 1 - x, 1 - y, 1 - z].

## supplementary crystallographic information

### Comment

The conformation of six-membered rings arranged by the bidentate coordination of pen (1,3-propanediamine) to transition metals has long been of theoretical interest (Gollogly & Hawkins, 1972). Despite this interest, only a limited number of such complexes have been structurally described. Because of their ability to undergo solid-state phase transitions, some nickel(II) complexes of bis(N-substituted-pen) have been studied in recent times (Mukherjee *et al.*, 1990; Pariya *et al.*, 1995; Ghosh *et al.*, 1997).

Squaric acid (H_2_C_4_O_4_) has been of much interest because of its cyclic structure and possible aromaticity. Recently, considerable progress has been made in the crystal engineering of multidimensional arrays and networks containing metal ions as nodes. Squaric acid is a useful tool for constructing crystalline architectures, due to its rigid, planar four membered ring skeleton, and its proton donating and accepting capabilities for hydrogen bonding (Bertolasi *et al.*, 2001; Reetz *et al.*, 1994; Lam & Mak, 2000; Zaman *et al.*, 2001; Mathew *et al.*, 2002). In addition, squaric acid has been studied for potetial application in xerographic photoreceptors, organic solar cells and optical recording (Liebeskind *et al.*, 1993; Seitz & Imming, 1992).

The asymmetric unit of the title compound contains one centrosymmetric cation, where Ni^II^ is located on a crystallographic inversion centre, one centrosymmetric anion and two uncoordinated water molecules (Fig.1 ), in which the bond lengths (Allen *et al.*,  1987) and angles are within normal ranges. In cation, the Ni^II^ is hexacoordinated by two O atoms of two water molecules in a *trans* order and by four N atoms of two pen ligands at the equatorial positions (Table 1). It is suggested that the *trans* geometry is preferred, when the amine ligand is more bulky. Thus, the coordination environment of Ni^II^ is a slightly distorted octahedral. Intramolecular O-H···O hydrogen bonds (Table 2) link the cation and anion through the water molecule, while intramolecular N-H···O hydrogen bonds (Table 2) link the cation and anion and cation and water molecule. The six-membered chelate ring (Ni1/N1/N2/C1-C3) is not planar, having total puckering amplitude, Q_T_, of 0.765 (3) Å and chair conformation [φ = 166.25 (3) and θ = 40.42 (3) °] (Cremer & Pople, 1975).

In the crystal structure, intermolecular O-H···O and N-H···O hydrogen bonds (Table 2) link the molecules into chains (Fig. 2), in which they may be effective in the stabilization of the structure.

### Experimental

For the preparation of the title compound, a solution of squaric acid (0.57 g, 5 mmol) in water (25 ml) was neutralized with sodium hydroxide (0.40 g, 10 mmol) and added dropwise with stirring to a solution of Ni(CH_3_COO)_2_.4(H_2_O) (1.24 g, 5 mmol) in water (25 ml) at 323 K. The solution immediately became suspension and was stirred for 2 h. Then, 1,3-propandiamine (0.74 g, 10 mmol) in methanol (10 ml) was added dropwise to the obtained suspension. The clear solution was stirred for 2 h, and then cooled to room temperature. The crystals formed were filtered and washed with water (10 ml) and methanol (1:1), then dried in air. Anal. Calcd. : C 28.12, H 7.55, N 13.12%; Found C 28.06, H 7.61, N 13.18%.

### Refinement

Atoms H1E, H1F, H4A, H4B, H5A and H5B (for H_2_O) were located in difference syntheses and refined isotropically. The remaining H atoms were positioned geometrically with N-H = 0.90 Å (for NH_2_) and C-H = 0.97 Å (for CH_2_) and constrained to ride on their parent atoms, with U_iso_(H) = 1.2U_eq_(C,N).

### Crystal data

**Table:**

[Ni(C_3_H_10_N_2_)_2_(H_2_O)_2_](C_4_O_4_)·4H_2_O	*F*(000) = 456.0
*M**_r_* = 427.09	*D*_x_ = 1.480 Mg m^−^^3^
Monoclinic, *P*2_1_/*c*	Mo *K*α radiation, λ = 0.71073 Å
Hall symbol: -P 2ybc	Cell parameters from 2204 reflections
*a* = 8.0429 (4) Å	θ = 2.2–28.0°
*b* = 9.1752 (5) Å	µ = 1.06 mm^−^^1^
*c* = 14.6510 (8) Å	*T* = 296 K
β = 117.570 (4)°	Plate, violet
*V* = 958.40 (9) Å^3^	0.75 × 0.45 × 0.05 mm
*Z* = 2	

### Data collection

**Table:**

Stoe IPDS II diffractometer	2204 independent reflections
Radiation source: sealed X-ray tube, 12 x 0.4 mm long-fine focus	2003 reflections with *I* > 2σ(*I*)
plane graphite	*R*_int_ = 0.020
Detector resolution: 6.67 pixels mm^-1^	θ_max_ = 27.5°, θ_min_ = 2.7°
w–scan rotation method	*h* = −10→10
Absorption correction: integration (*X-RED32*; Stoe & Cie, 2002)	*k* = −11→11
*T*_min_ = 0.638, *T*_max_ = 0.949	*l* = −19→17
7288 measured reflections	

### Refinement

**Table:**

Refinement on *F*^2^	Primary atom site location: structure-invariant direct methods
Least-squares matrix: full	Secondary atom site location: difference Fourier map
*R*[*F*^2^ > 2σ(*F*^2^)] = 0.021	Hydrogen site location: inferred from neighbouring sites
*wR*(*F*^2^) = 0.054	H atoms treated by a mixture of independent and constrained refinement
*S* = 1.06	*w* = 1/[σ^2^(*F*_o_^2^) + (0.0282*P*)^2^ + 0.2118*P*] where *P* = (*F*_o_^2^ + 2*F*_c_^2^)/3
2204 reflections	(Δ/σ)_max_ < 0.001
139 parameters	Δρ_max_ = 0.29 e Å^−^^3^
0 restraints	Δρ_min_ = −0.17 e Å^−^^3^

### Special details

**Table:**

Geometry. All e.s.d.'s (except the e.s.d. in the dihedral angle between two l.s. planes) are estimated using the full covariance matrix. The cell e.s.d.'s are taken into account individually in the estimation of e.s.d.'s in distances, angles and torsion angles; correlations between e.s.d.'s in cell parameters are only used when they are defined by crystal symmetry. An approximate (isotropic) treatment of cell e.s.d.'s is used for estimating e.s.d.'s involving l.s. planes.
Refinement. Refinement of *F*^2^ against ALL reflections. The weighted *R*-factor *wR* and goodness of fit *S* are based on *F*^2^, conventional *R*-factors *R* are based on *F*, with *F* set to zero for negative *F*^2^. The threshold expression of *F*^2^ > σ(*F*^2^) is used only for calculating *R*-factors(gt) *etc*. and is not relevant to the choice of reflections for refinement. *R*-factors based on *F*^2^ are statistically about twice as large as those based on *F*, and *R*- factors based on ALL data will be even larger.

### Fractional atomic coordinates and isotropic or equivalent isotropic displacement parameters (Å^2^)

**Table:**

	*x*	*y*	*z*	*U*_iso_*/*U*_eq_	
Ni1	0.5000	0.5000	0.5000	0.02232 (7)	
O1	0.64732 (15)	0.39000 (12)	0.43015 (8)	0.0383 (2)	
H1E	0.713 (3)	0.432 (2)	0.4113 (14)	0.051 (5)*	
H1F	0.692 (3)	0.315 (3)	0.4515 (15)	0.063 (6)*	
O2	0.73874 (13)	1.09844 (11)	0.49933 (8)	0.0414 (2)	
O3	0.86500 (14)	0.77497 (10)	0.46843 (9)	0.0434 (2)	
O4	0.34274 (18)	0.07964 (14)	0.36302 (9)	0.0489 (3)	
H4A	0.455 (3)	0.082 (2)	0.3910 (16)	0.067 (6)*	
H4B	0.317 (3)	0.029 (3)	0.401 (2)	0.075 (7)*	
O5	0.85562 (18)	0.53525 (13)	0.35580 (11)	0.0462 (3)	
H5A	0.871 (3)	0.611 (3)	0.3859 (17)	0.066 (6)*	
H5B	0.803 (3)	0.550 (3)	0.2975 (19)	0.072 (8)*	
N1	0.24635 (14)	0.41813 (12)	0.38210 (8)	0.0317 (2)	
H1A	0.2548	0.3203	0.3845	0.038*	
H1B	0.1547	0.4421	0.3983	0.038*	
N2	0.47942 (14)	0.69084 (11)	0.41632 (8)	0.0294 (2)	
H2A	0.4236	0.7588	0.4372	0.035*	
H2B	0.5970	0.7221	0.4355	0.035*	
C1	0.1833 (2)	0.46195 (17)	0.27461 (10)	0.0423 (3)	
H1C	0.0568	0.4263	0.2326	0.051*	
H1D	0.2640	0.4173	0.2497	0.051*	
C2	0.1860 (2)	0.62587 (16)	0.26262 (11)	0.0438 (3)	
H2C	0.1144	0.6502	0.1902	0.053*	
H2D	0.1242	0.6710	0.2987	0.053*	
C3	0.3806 (2)	0.68931 (16)	0.30294 (10)	0.0406 (3)	
H3A	0.4520	0.6320	0.2776	0.049*	
H3B	0.3718	0.7880	0.2774	0.049*	
C4	0.88219 (16)	1.04411 (13)	0.49952 (9)	0.0269 (2)	
C5	0.93844 (16)	0.89777 (13)	0.48539 (9)	0.0271 (2)	

### Atomic displacement parameters (Å^2^)

**Table:**

	*U*^11^	*U*^22^	*U*^33^	*U*^12^	*U*^13^	*U*^23^
Ni1	0.02176 (11)	0.02133 (11)	0.02386 (11)	0.00055 (7)	0.01055 (8)	0.00121 (7)
O1	0.0460 (6)	0.0308 (5)	0.0522 (6)	0.0070 (4)	0.0346 (5)	0.0041 (4)
O2	0.0319 (5)	0.0362 (5)	0.0643 (7)	0.0049 (4)	0.0291 (5)	0.0006 (5)
O3	0.0453 (5)	0.0286 (5)	0.0636 (7)	−0.0116 (4)	0.0314 (5)	−0.0089 (4)
O4	0.0416 (6)	0.0539 (7)	0.0491 (6)	−0.0045 (5)	0.0191 (5)	0.0039 (5)
O5	0.0530 (7)	0.0407 (6)	0.0538 (7)	−0.0009 (5)	0.0321 (6)	−0.0024 (5)
N1	0.0279 (5)	0.0315 (5)	0.0311 (5)	−0.0026 (4)	0.0097 (4)	−0.0008 (4)
N2	0.0309 (5)	0.0258 (5)	0.0317 (5)	0.0005 (4)	0.0147 (4)	0.0031 (4)
C1	0.0455 (8)	0.0434 (7)	0.0282 (6)	−0.0033 (6)	0.0089 (6)	−0.0053 (5)
C2	0.0453 (8)	0.0452 (8)	0.0278 (6)	0.0055 (6)	0.0058 (6)	0.0062 (6)
C3	0.0517 (8)	0.0407 (7)	0.0322 (6)	0.0026 (6)	0.0219 (6)	0.0086 (5)
C4	0.0251 (5)	0.0269 (5)	0.0296 (6)	0.0008 (4)	0.0134 (4)	0.0015 (4)
C5	0.0269 (5)	0.0263 (5)	0.0290 (6)	−0.0021 (4)	0.0138 (4)	−0.0006 (4)

### Geometric parameters (Å, °)

**Table:**

Ni1—O1^i^	2.1429 (9)	C1—N1	1.4695 (17)
Ni1—N1^i^	2.1090 (10)	C1—C2	1.516 (2)
Ni1—N2^i^	2.0997 (10)	C1—H1C	0.9700
O1—Ni1	2.1429 (9)	C1—H1D	0.9700
O1—H1E	0.80 (2)	C2—C3	1.511 (2)
O1—H1F	0.77 (2)	C2—H2C	0.9700
O4—H4A	0.80 (2)	C2—H2D	0.9700
O4—H4B	0.82 (3)	C3—N2	1.4728 (16)
O5—H5A	0.80 (2)	C3—H3A	0.9700
O5—H5B	0.77 (2)	C3—H3B	0.9700
N1—Ni1	2.1090 (10)	C4—O2	1.2557 (15)
N1—H1A	0.9000	C4—C5^ii^	1.4557 (16)
N1—H1B	0.9000	C4—C5	1.4619 (17)
N2—Ni1	2.0997 (10)	C5—O3	1.2427 (15)
N2—H2A	0.9000	C5—C4^ii^	1.4557 (16)
N2—H2B	0.9000		
			
O1—Ni1—O1^i^	180.00 (5)	C3—N2—Ni1	120.41 (8)
N1^i^—Ni1—N1	180.0	C3—N2—H2A	107.2
N1^i^—Ni1—O1	91.14 (4)	C3—N2—H2B	107.2
N1—Ni1—O1	88.86 (4)	H2A—N2—H2B	106.9
N1^i^—Ni1—O1^i^	88.86 (4)	N1—C1—C2	112.30 (11)
N1—Ni1—O1^i^	91.14 (4)	N1—C1—H1C	109.1
N2^i^—Ni1—O1	88.54 (4)	C2—C1—H1C	109.1
N2—Ni1—O1	91.46 (4)	N1—C1—H1D	109.1
N2^i^—Ni1—O1^i^	91.46 (4)	C2—C1—H1D	109.1
N2—Ni1—O1^i^	88.54 (4)	H1C—C1—H1D	107.9
N2^i^—Ni1—N2	180.0	C3—C2—C1	113.88 (12)
N2^i^—Ni1—N1^i^	91.94 (4)	C3—C2—H2C	108.8
N2—Ni1—N1^i^	88.06 (4)	C1—C2—H2C	108.8
N2^i^—Ni1—N1	88.06 (4)	C3—C2—H2D	108.8
N2—Ni1—N1	91.94 (4)	C1—C2—H2D	108.8
Ni1—O1—H1E	122.4 (14)	H2C—C2—H2D	107.7
Ni1—O1—H1F	118.8 (15)	N2—C3—C2	111.42 (11)
H1E—O1—H1F	108.4 (19)	N2—C3—H3A	109.3
H4A—O4—H4B	104 (2)	C2—C3—H3A	109.3
H5A—O5—H5B	109 (2)	N2—C3—H3B	109.3
Ni1—N1—H1A	107.3	C2—C3—H3B	109.3
Ni1—N1—H1B	107.3	H3A—C3—H3B	108.0
C1—N1—Ni1	120.23 (9)	O2—C4—C5^ii^	134.42 (12)
C1—N1—H1A	107.3	O2—C4—C5	135.11 (12)
C1—N1—H1B	107.3	C5^ii^—C4—C5	90.46 (9)
H1A—N1—H1B	106.9	O3—C5—C4^ii^	135.11 (12)
Ni1—N2—H2A	107.2	O3—C5—C4	135.35 (12)
Ni1—N2—H2B	107.2	C4^ii^—C5—C4	89.54 (9)
			
C1—N1—Ni1—O1	−63.90 (10)	N1—C1—C2—C3	72.93 (17)
C1—N1—Ni1—O1^i^	116.10 (10)	C1—C2—C3—N2	−73.63 (16)
C1—N1—Ni1—N2^i^	−152.47 (10)	C2—C3—N2—Ni1	52.35 (14)
C1—N1—Ni1—N2	27.53 (10)	O2—C4—C5—O3	−0.2 (3)
C3—N2—Ni1—O1	60.31 (10)	C5^ii^—C4—C5—O3	179.50 (19)
C3—N2—Ni1—O1^i^	−119.69 (10)	O2—C4—C5—C4^ii^	−179.72 (18)
C2—C1—N1—Ni1	−50.38 (16)	C5^ii^—C4—C5—C4^ii^	0.0

Symmetry codes: (i) −*x*+1, −*y*+1, −*z*+1; (ii) −*x*+2, −*y*+2, −*z*+1.

### Hydrogen-bond geometry (Å, °)

**Table:**

*D*—H···*A*	*D*—H	H···*A*	*D*···*A*	*D*—H···*A*
N1—H1B···O5^iii^	0.90	2.35	3.1715 (16)	152
N2—H2A···O2^iv^	0.90	2.33	3.2174 (14)	170
O1—H1F···O2^v^	0.77 (2)	2.08 (2)	2.8345 (15)	165 (2)
O4—H4A···O2^v^	0.80 (2)	2.10 (2)	2.8765 (16)	165 (2)
O4—H4B···O2^i^	0.82 (3)	2.07 (3)	2.8965 (16)	178 (2)
O5—H5B···O4^vi^	0.77 (2)	2.10 (3)	2.8730 (19)	177 (2)
N1—H1A···O4	0.90	2.38	3.2442 (17)	160
N2—H2B···O3	0.90	2.04	2.9333 (14)	174
O1—H1E···O5	0.80 (2)	1.93 (2)	2.7311 (16)	175.8 (19)
O5—H5A···O3	0.80 (2)	1.94 (2)	2.7296 (16)	166 (2)

Symmetry codes: (iii) *x*−1, *y*, *z*; (iv) −*x*+1, −*y*+2, −*z*+1; (v) *x*, *y*−1, *z*; (i) −*x*+1, −*y*+1, −*z*+1; (vi) −*x*+1, *y*+1/2, −*z*+1/2.
